# Agent-based model for simulating microbial movement with a preferred target for agricultural biologicals

**DOI:** 10.7717/peerj.21641

**Published:** 2026-09-08

**Authors:** Shuai Yuan, Chien-En Liao, Chia-An Cho, Ko-Hsuan Chen, Steven H. Wu

**Affiliations:** 1 Department of Agronomy, National Taiwan University, Taipei, Taiwan; 2 Biodiversity Research Center, Academia Sinica, Taipei, Taiwan

**Keywords:** Agent-based model, Agricultural biological, Simulation, Microbial resource preference, Microbial population dynamics

## Abstract

Agricultural biologicals utilize beneficial microorganisms to enhance crop health and productivity, representing a rapidly growing sector of sustainable agriculture, offering environmentally friendly alternatives to synthetic chemicals. A key determinant of their efficacy is the ability of inoculated microbes to navigate through complex soil environments and reach plant roots, a process that is challenging to study empirically. To address this, we developed and validated a flexible Agent-Based Model (ABM) that simulates microbial spatial dynamics in soil. Each agent is characterized by unique attributes, including age, location, and parameters describing its behavior and interactions with the surroundings. Microbial behavior is governed by two core mechanisms: (1) logistic-based birth-death processes for population dynamics, and (2) directional preference for movement. Directional preference is driven by resource attraction toward plant root exudates, modeled with an attraction strength parameter (σ), and density-dependent interactions, modeled with a repulsion intensity parameter (γ). Validation used both *in silico* simulation and laboratory growth experiments. Simulations confirmed that the ABM reproduced theoretical population dynamic curves and realistic environmental responses. Lower σ values produced tight clustering around resources, while higher γ values resulted in avoidance of high density area. Laboratory experiments confirmed preferential fungal growth toward nutrient sources, with simulated results best matching observed growth patterns at σ values between 2.5 and 3.5. This ABM provides a computationally tractable framework for investigating microbial behavior in agricultural systems, with potential applications in screening and optimizing microbial candidates for agricultural biological products.

## Introduction

Agriculture has faced multiple challenges in recent years, including ensuring food security for a growing global population while minimizing environmental impact and protecting human health (Mishra & Dash, 2014; Rehman et al., 2022). These challenges have driven a shift toward sustainable agricultural practices, which include growing interest in replacing synthetic chemical products with beneficial microorganisms to enhance soil nutrient availability, control plant diseases, and mitigate environmental stresses (Elnahal et al., 2022; Araújo et al., 2023). This shift toward agricultural biological solutions represents a fundamental change that proposes promising alternatives by moving away from intensive chemical usage while providing a sustainable environment for the future (Shahwar et al., 2023).

Agricultural biologicals encompass a diverse range of products and technologies that utilize natural or biological processes to improve agricultural productivity, sustainability, and resilience (Sharma, 2014; Santos et al., 2024). These products include beneficial microorganisms such as bacteria and fungi, natural molecules, plant extracts, enzymes, and other biologically-derived substances. These products are further classified into three major categories: biofertilizers, biostimulants, and biopesticides. Biofertilizers enhance nutrient availability and uptake by plants, while biostimulants promote plant growth and stress tolerance. Biopesticides utilize biological agents to control pests and diseases. Each category has distinct but complementary roles in overall sustainable crop production (Rajput et al., 2019; Bamdad et al., 2022; Chaudhary et al., 2022). Current application methods for agricultural microbial products include foliar application, seed treatment, and soil drenching, where liquid products are applied directly to the soil surface near the crop (Ahmad et al., 2024).

Despite these promising results, one major challenge is the ability of microorganisms to navigate successfully through complex soil environments *via* chemotactic responses to reach target sites such as plant roots or the rhizosphere, and subsequently establish beneficial plant-microbe interactions (Yang et al., 2024). This navigation challenge is complicated by the extraordinary complexity of soil microbial communities, which can contain millions to billions of microorganisms per gram of soil, comprised of thousands of different species with diverse ecological functions and interactions (Torsvik & Øvreås, 2002). Understanding how inoculated beneficial microbes move through and interact with the complex microbial landscape in the soil is crucial for optimizing the efficacy of agricultural biologicals and advancing sustainable agriculture practices.

Two key mechanisms that govern the microbial movement in soil environments and must be incorporated into the simulation framework are population dynamics and directional preference. Microbial population dynamics encompass both birth and death processes, with net population change representing the balance between these competing mechanisms. These dynamics are driven by a range of biological, chemical, and physical factors, including temperature, pH, nutrient availability, and oxygen levels (Mitchell et al., 2004; König et al., 2020). Several mathematical models have been developed to describe microbial population dynamics. The Monod model describes growth in nutrient-limited environments, where net population change is proportional to available nutrient concentrations (Lobry et al., 1992). The logistic model describes the decrease in growth rate as the population approaches the environmental carrying capacity (Tsoularis & Wallace, 2002). More complex models include the Gompertz, Baranyi, and Buchanan models, which incorporate additional parameters to capture different population dynamic phases and responses to environmental conditions (Buchanan, Whiting & Damert, 1997). Directional preference in soil microbes is driven by responses to environmental gradients, including plant root exudates that create chemical signals attracting beneficial microbes toward the rhizosphere through chemotaxis (Abu-Ashour et al., 1994; Mitchell et al., 2004; Canarini et al., 2019). Additionally, density-dependent competitive pressures promote dispersal away from overcrowded regions (Hibbing et al., 2010). This dual influence of resource attraction and density-dependent repulsion creates complex spatial patterns that ultimately determine the success of plant-microbe interactions.

The existing modeling approaches often use differential equation-based frameworks to simulate microbial communities. Nevertheless, these approaches face inherent difficulty in building comprehensive mathematical modeling systems that account for all environmental variables and microbial interactions, particularly when underlying mechanisms are not fully understood (Song et al., 2014; Koshy-Chenthittayil et al., 2021; Nagarajan, Ni & Lu, 2022).

An Agent-Based Model (ABM) is a computational approach that represents a system as a collection of individual decision-making units called agents. Each agent has its own attributes and follows a set of predefined rules that enable autonomous responses to environmental changes and interactions with other agents (Bonabeau, 2002; Nagarajan, Ni & Lu, 2022). Through these interactions, ABMs capture both localized microbial dynamics at the individual level and overall population level outcomes. Unlike differential equation-based approaches, ABMs can achieve this without requiring full specification of underlying mathematical models (An, 2012). Furthermore, ABM provides *in silico* experimentation to explore scenarios that are impractical or impossible to implement in laboratory, greenhouse, or field settings. This capability is particularly valuable for studying agricultural biologicals, where outcomes depend on numerous interacting factors including inoculation methods, environmental conditions, and management practices.

ABMs have been successfully applied across diverse fields, from epidemiology and ecology to social networks and financial markets (Will et al., 2020), demonstrating the power of this approach to capture emergent behaviors in complex systems. In epidemiology, ABMs have been used to simulate COVID-19 transmission dynamics by modeling unique characteristics of individual citizens and their interactions (Cuevas, 2020; Kerr et al., 2021). Despite the wide range of applications across diverse fields, the use of ABMs in soil microbial systems in the context of agricultural biologicals remains limited, representing an important gap in the current modeling landscape.

The primary objective of this study is to both develop and systematically validate an ABM that simulates the spatial dynamics of microbial communities in soil environments, with particular relevance to agricultural biological applications. The model aims to improve understanding of how inoculated microbes navigate through complex soil microbial communities and respond to plant-derived chemical signals. The model integrates logistic-based birth-death processes for population dynamics with directional preference mechanisms governed by resource attraction toward plant exudates and density-dependent repulsion from neighboring microbes. This model specifically addresses the critical assumption underlying soil drenching application methods, where inoculated microorganisms need to successfully navigate through complex, microbe-rich soil environments to reach and colonize target plant roots. Validation was conducted through systematic simulation studies evaluating individual model components and their integration into comprehensive scenarios mimicking real-world agricultural conditions, as well as laboratory experiments examining fungal growth patterns toward nutrient sources on agar plates. The model is publicly available on GitHub, providing a flexible and extensible computational platform that can be tailored to diverse research questions in agricultural microbiology.

## Materials and Methods

### Research design and procedures

The study was conducted in three sequential stages: (1) development of the Agent-Based Model (ABM), (2) simulation analyses to validate and characterize model behavior, and (3) laboratory validation of the model behaviors using fungal growth experiments. In Stage 1, the ABM was developed to simulate microbial population dynamics and directional movement through soil environments toward plant root targets. In Stage 2, simulation analyses were performed in three progressive steps: population dynamics validation, directional preference parameter validation, and combined comprehensive simulations designed to mimic real-world agricultural scenarios. In Stage 3, fungal growth experiments provided empirical observations against the simulated results. This enables identification of parameter combinations that explain observed biological behavior.

The Agent-Based Model (ABM) was developed to simulate microbial population dynamics and directional preference through soil environments toward plant root targets. The model incorporated birth-death mechanisms for population dynamics and directional preference in response to plant root exudates and microbial interactions with surrounding microbes. The model addressed a key assumption for the soil drenching application method, where the beneficial microorganisms applied through soil drenching have to successfully navigate through complex soil environments to reach target sites such as plant roots. There are three key assumptions that make this simulation model computationally feasible. First, due to the billions of microbes in soil environments, it is computationally impractical to represent each microbe as an individual agent. Therefore, each agent represents a small colony of microbes with similar behaviors that operate as a single functional unit. Second, the model assumes relatively homogeneous soil environment where microbes can move freely without physical barriers. Third, the microbes only interact with their local neighbors, and not all microorganisms in the soil. These assumptions enable the model to simulate realistic microbial behavior while maintaining population-level dynamics modeling and remaining computationally tractable.

### Agent-based model (ABM)

The ABM is an *in silico* simulation approach that utilizes iterative time steps to model complex interactions. This methodology captures dynamic processes by breaking time into discrete intervals instead of continuous time. It allows simulation of individual agents and their interactions over time to eventually capture overall system-level dynamics. In reality, each iteration represents a reproduction interval of a microbial organism, typically 10–20 min under optimal conditions (Zwietering et al., 1990). The ABM is made up of a large number of agents, each defined by specific attributes. Each agent in the model is characterized by five fundamental attributes that define its essential properties. These attributes are agent ID, age, parent, location, and functional class. The “agent ID” attribute serves as a unique identifier for each agent, enabling tracking of individual agents throughout the simulation. The “age” attribute represents the number of iterations an agent has undergone since its creation, starting from 0. The “parent” attribute tracks the lineage of each agent by recording its parent’s ID. It provides genealogical information that can track the population dynamics and inheritance patterns. The “location” attribute specifies the agent’s current position using x and y coordinates in a two-dimensional simulated environment. Agent positions are updated each iteration and are influenced by resource attraction factors and surrounding microbes. The “functional class” attribute differentiates microbial species or functional groups. This represents a fundamental feature in the ABM that takes into account microbial heterogeneity and behaviors. Each class possesses several distinct attributes that determine specific behaviors, including birth and death coefficients, parameters related to spatial patterns, and interactions and responses to the surrounding environment. Different classes can exhibit varying degrees of resource attraction, diverse movement speeds, or even stationary behavior. This framework allows for complex interactions between classes and enables the construction of comprehensive models that capture diverse microbial behaviors. This includes representation of both inoculated beneficial microbes and native background soil microbial communities with distinct behaviors. The implementation facilitates the modeling of realistic microbial community interactions and population dynamics.

**Figure 1:**
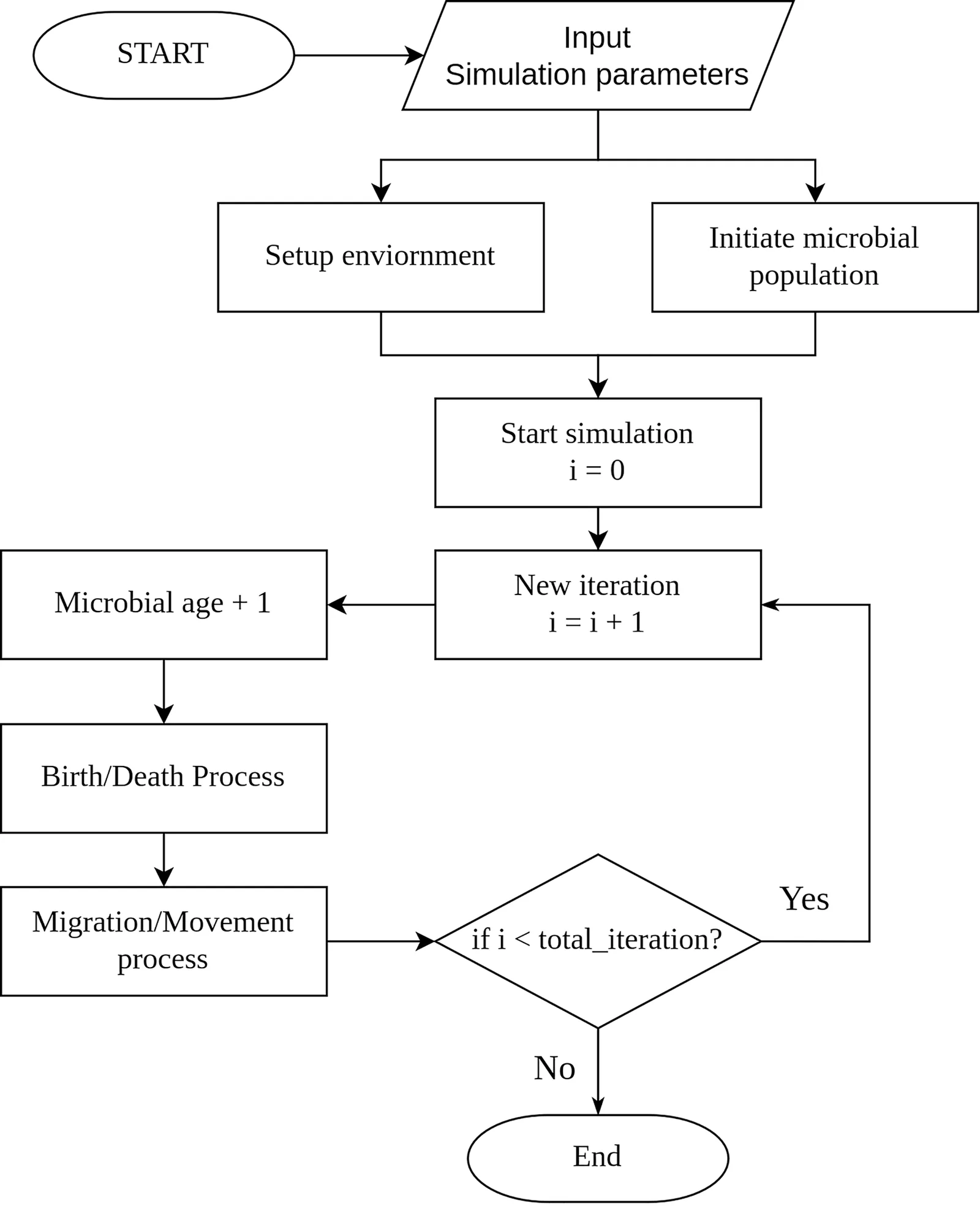
Overview of the agent-based model simulation process. The simulation initializes with randomly distributed agents (microbes) and environment. Each iteration involves three sequential steps: (1) age increment, (2) changes in population dynamics, and (3) movement based on resource preference.

The simulation begins with an initial setup where a predetermined number of agents are randomly distributed within a designated area, each assigned the five fundamental attributes described above, along with resource attraction sources in the background environment. During each iteration, agents follow a sequential process: (1) age increments by one unit, (2) changes in microbial population size *via* birth and death processes, and (3) directional preference determined by resource attraction and density of surrounding microbes. The simulation repeats and operates through these iterative cycles. This simulation framework enables the simulation of realistic scenarios where beneficial microbes must compete with existing soil communities while navigating toward plant targets through chemotactic responses to root exudates. The overview of the simulation process is summarized in Fig. 1.

#### Population dynamics

The population dynamics of the model are based on the hypotheses that a carrying capacity exists, which is the maximum population size that the environment can sustain. This is the result of multiple factors, including space and resource constraints (Mahanty et al., 2017). Equation (1) describes the theoretical logistic growth model using a differential equation (Tsoularis & Wallace, 2002):

(1) $$\displaystyle{{dN} \over {dt}} = \; \rho N\left( {\displaystyle{{K - N} \over K}} \right)$$

where *N* represents the current population size, ρ stands for the intrinsic growth rate of the microbe, *t* indicates the age, and *K* refers to the total carrying capacity of the environment. At the initial stages of growth, when *N* is relatively small, the population undergoes an exponential growth phase that is proportional to the growth rate ρ. As the population continues to grow and approaches the carrying capacity, the rate of population increase starts to decline.

The ABM approximates biologically realistic population dynamics through birth and death coefficients, which are used to simulate the probabilities of creating or removing an agent from the environment. The probabilities for an agent to reproduce or die during an iteration are presented in Eqs. (2) and (3):

(2) $$P\left( {birth} \right) = min\left\{ {1,\; b\left( {1 - \displaystyle{N \over K}} \right)} \right\}$$

(3) $$P\left( {death} \right) = min\left\{ {1,\; d\left( {\displaystyle{N \over K}} \right)} \right\}$$

where *b* and *d* represent the proportional birth and death coefficients, respectively. These equations rely on proportional relationships based on the current population size and the carrying capacities. The probability of birth is maximized when the population N is approaching 0, and the maximum death probability is achieved when the population N is approaching K.

#### Directional preference mechanisms

Microbes’ movement and directional growth through soil is considerably more complex and largely untraceable under real-world conditions (Zhu et al., 2017). The aim of this study is to construct a model based on two influential factors: resource attraction and surrounding density. The resource attraction factor simulates the attraction of root exudates, as plant-associated microbes tend to move towards nutrient-rich areas and interact with plants. The surrounding density factor simulates competition among surrounding microbes in the soil. The location of agents is represented in a two-dimensional coordinate system, with microbes tracked through changes in x and y coordinates. Position changes are determined by the agent’s movement speed and direction (angle). For instance, if a microbe’s initial position is $\left( {a,\; b} \right)$, its speed is *s*, and it moves along an angle θ, then its updated position will be calculated by Eq. (4):

(4) $$\left( {a + cos\left( \theta \right)*s,\; b + sin\left( \theta \right)*s} \right)$$

##### Movement speed

The “movement speed” attribute determines how far an agent can move or grow in the simulation environment during one iteration. It follows a uniform distribution to ensure that agents do not move the same distance each iteration. To simplify the model, the measurements for speed and distance values are not assigned specific units, and these parameters are interpreted as relative speed and distance rather than exact values. In reality, microbial motility varies greatly and can reach speeds of up to 60 micrometers per second (Bente et al., 2020).

##### Resource attraction factor

**Figure 2:**
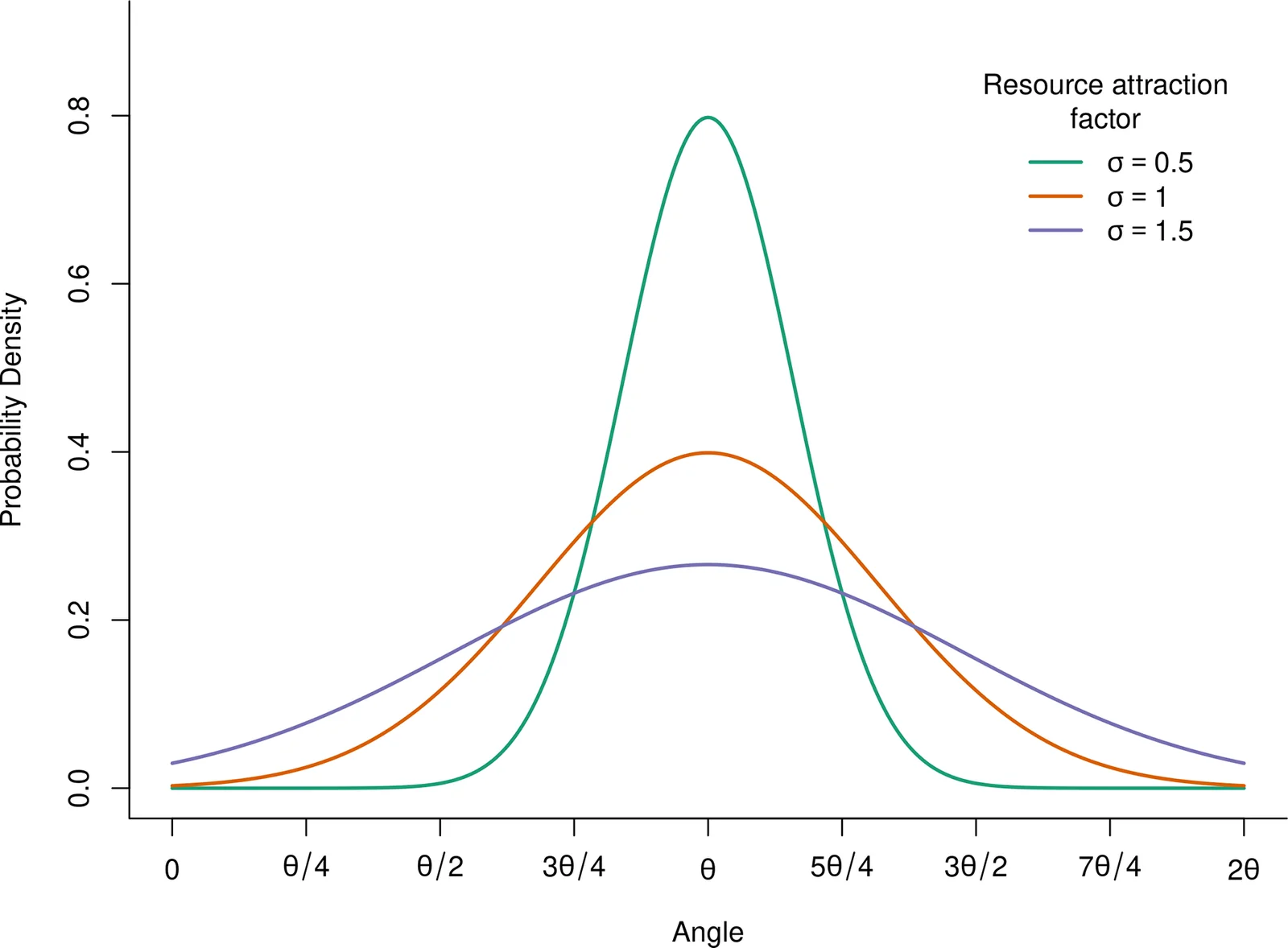
Resource-based directional preference mechanism. Illustrates the probability distribution of preference directions when a resource is located at angle θ relative to an agent. The curve follows a normal distribution with mean equal to θ. A small attraction strength parameter (σ) increases the attractiveness of the resource by increasing the preference movement within a narrow range of directions around this angle.

To quantitatively describe microbial preference towards resources, a normal distribution of the moving angle reflects the tendency of microbes to move towards resource-rich areas, with the mean value determined by the angle between the agent and the resource, denoted as θ. In this study, resource locations are assumed to be fixed and do not take into account the diffusion of plant exudates in the soil. For each angle *x* within the range of [0, 2π), the probability of movement for each angle is calculated using Eq. (5):

(5) $$r\left( {x|\theta ,\sigma } \right) = {1 \over {\sigma \sqrt {2\pi } }}\; {e^{ - {1 \over 2}{{\left( {{{x - \theta } \over \sigma }} \right)}^2}}}\;$$

where σ is the attraction strength parameter, which determines the intensity of the resource attraction. A smaller σ represents a stronger level of attraction to the resource, resulting in a higher probability of movement within a narrow range of directions around the target angle, as illustrated in Fig. 2.

##### Surrounding density factor

**Figure 3:**
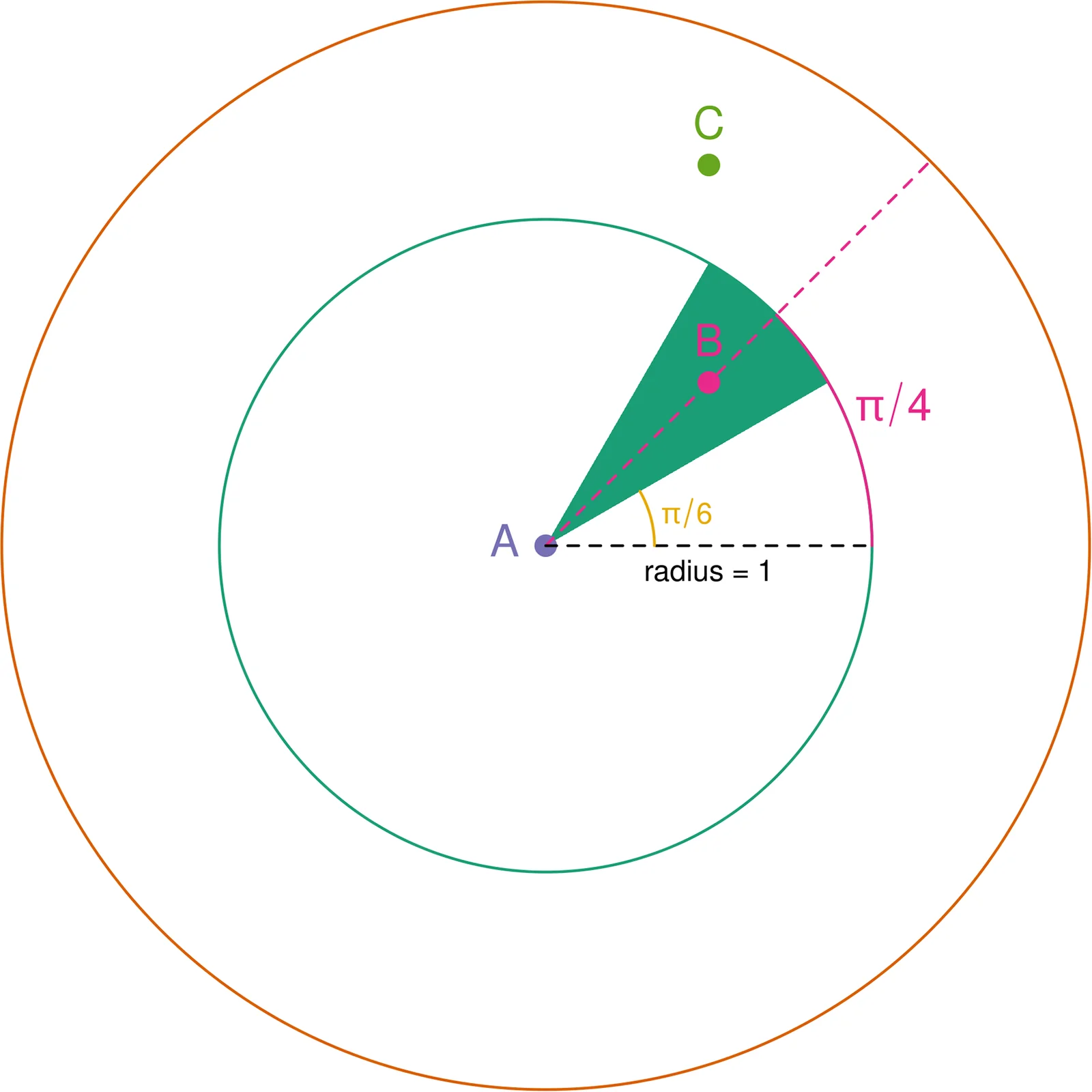
Density-based directional preference mechanism. Illustration of the detection zone for agent A with a detection radius of ρ = 1 and an angular range of α = π/12 (15°) degrees. Agent B is at a θ = π/4 (45°) degree from agent A, and the green shaded area, within the π/4 ± π/12 (45° ± 15°), represents the detection zone of agent A. Within the shaded area, agent A can detect neighboring agents that influence its directional preference. Agents beyond the detection radius do not affect each other, such as agent C.

The directional preference of microbes is also influenced by local cell density, as microbes prefer to seek less crowded environments to reduce resource competition (Du et al., 2021). This behavior is observed in biofilm dispersion, where cells detach from biofilms and migrate to surrounding areas (Rumbaugh & Sauer, 2020). This interaction with the surrounding density is governed by a detection system for each agent consisting of three key parameters: detection radius (ρ), detection angle (α), and repulsion intensity parameter (γ). The detection radius and angle define a detection zone in which an agent can sense neighboring agents. For any given direction θ, the detection zone forms a sector of a circle with radius ρ and central angle ranging from $\theta - \alpha$ to $\theta + \alpha$ degrees. The probability of moving toward any given direction is inversely proportional to the local density, which is calculated by Eq. (6):

(6) $$d\left( x \right) = \displaystyle{1 \over {{\gamma ^{log\left( n \right)}}}}$$

where *n* is the local density represented by the total number of agents within the detection zone, and γ is the repulsion intensity parameter. A larger value of γ corresponds to stronger avoidance of crowded areas. By setting the radius ρ to 0, or γ to 1, this effectively removes the density effect and the model reduces to a simpler form that only considers the resource attraction factor. This mechanism ensures that agents preferentially move toward less populated areas, effectively simulating the density-avoidance behavior observed in microbial systems. Figure 3 illustrates the density-based probabilistic directional preference mechanism with three agents (A, B, and C) using parameters: detection radius ρ=1, angular range $\alpha = \pi /12$ (15°).

The comprehensive directional preference mechanism for each agent is determined by combining both resource attraction and surrounding density factors. For each microbial agent, the probability of remaining stationary (*i.e*., at the same location) was set to s=0.1. When movement occurs with probability of $1 - s\; = \; 0.9$, the angle of movement is determined by integrating both resource attraction and local surrounding density. The probability for each angle is calculated as the product of the equations described above, with the resulting probability distribution normalized across all possible angles.

### ABM model validation

Model validation was conducted through two complementary components: simulation validation examining model mechanisms, and biological validation using laboratory experiments to provide empirical benchmarks for comparison.

#### Simulation validation analyses

To validate the birth-death mechanisms in the ABM, a systematic grid search was conducted across different levels of birth and death coefficients: 0.1, 0.3, 0.7, and 0.9. Although population growth is inherently influenced by initial population size and carrying capacity, these parameters were held constant at 10 and 10,000, respectively.

To investigate the effect of the directional preference, the birth and death mechanisms were removed from the following simulation analyses. To test the attraction strength parameter (σ), 500 microbes were initially distributed randomly within a small area, and the resource was located outside this small area. This setup effectively mimics agricultural scenarios where microbial products are applied to soil, and microbes are required to navigate toward plant roots. A range of attraction strength parameters (σ) was tested: 0.1, 0.5, 1, 1.5, 2, 3, 5, and 10. The simulation process was monitored across all iterations.

Three parameters quantify density effects on microbial directional preference: detection radius (ρ), detection angle ( α), and repulsion intensity parameter (γ). The effects of these density related parameters are difficult to verify in unrestricted simulations due to the stochastic nature of ABMs. When agents cluster tightly, density effects may appear uniform across all directions and masking the effect. On the other hand, when agents undergo repulsion based preference, the influences on probabilistic motion may be masked due to apparent randomness. Therefore, a highly controlled setup was designed to validate the density based preference model. Five hundred stationary agents (green dots) representing background microbes in the soil were distributed within a central circular area, and these agents have their movement speed fixed at 0. There were 180 active and mobile agents (orange dots) positioned around the background agents to test the effect of various repulsion parameters. Simulation analyses were conducted examining multiple levels of repulsion intensity parameter γ = 0.2, 0.5, 0.75, 1, 1.5, 2, and 5. The value for other parameters was fixed at detection radius = 2, and detection angle = 45°.

The overall flowchart illustrating all mechanisms for the ABM is outlined in Fig. 1. The simulation analyses attempt to mimic agricultural scenarios where beneficial microbial treatments are inoculated into soil already populated with native microorganisms. These native microbes interact and compete with the microbial treatments, potentially hindering the treatments’ movement toward targets such as plant roots. The comprehensive evaluation of the ABM aims to simulate the behavior of two different groups of microbes under complex and realistic scenarios.

Simulations were initialized with 100 active agents with resource preferences randomly distributed within a central area, and 1,000 background agents without resource preferences dispersed around the large surrounding area. The resource was placed five units away from the center, and only active microbes had a preference for moving toward the resource. These background agents represent microbes already existing in the soil and have reached population equilibrium before inoculation with the active microbial treatment. The active agents represent microbial treatments that seek and move toward the resources. To maintain a stable overall population size in this simulation, the population dynamics parameters in the model were set with the birth coefficient (b) at 0.3, the death coefficient (d) at 0.1, and the environmental carrying capacity for active microbes (K) at 10,000. Other parameters, such as detection radius (ρ) and angle (α), were fixed at 2° and 45°, respectively. Multiple combinations of the attraction strength parameter (σ = {0.5, 1, 1.5}) and the repulsion intensity parameter (γ = {1, 1.5, 2}) were used in the simulation studies to investigate the effects of microbial behavior.

#### Biological validation analyses

Both fungi and bacteria are commonly used microorganisms in agricultural biologicals. Therefore, fungal growth experiments were conducted to validate key aspects of our simulation model. The experiments investigated growth patterns of fungal hyphae on bidirectional growth plates and examined how fungi detect and respond to food sources in their environment through chemotaxis. Growth patterns were compared with simulated results from the ABM to identify potential underlying parameter combinations that could explain the observed growth behavior.

**Figure 4:**
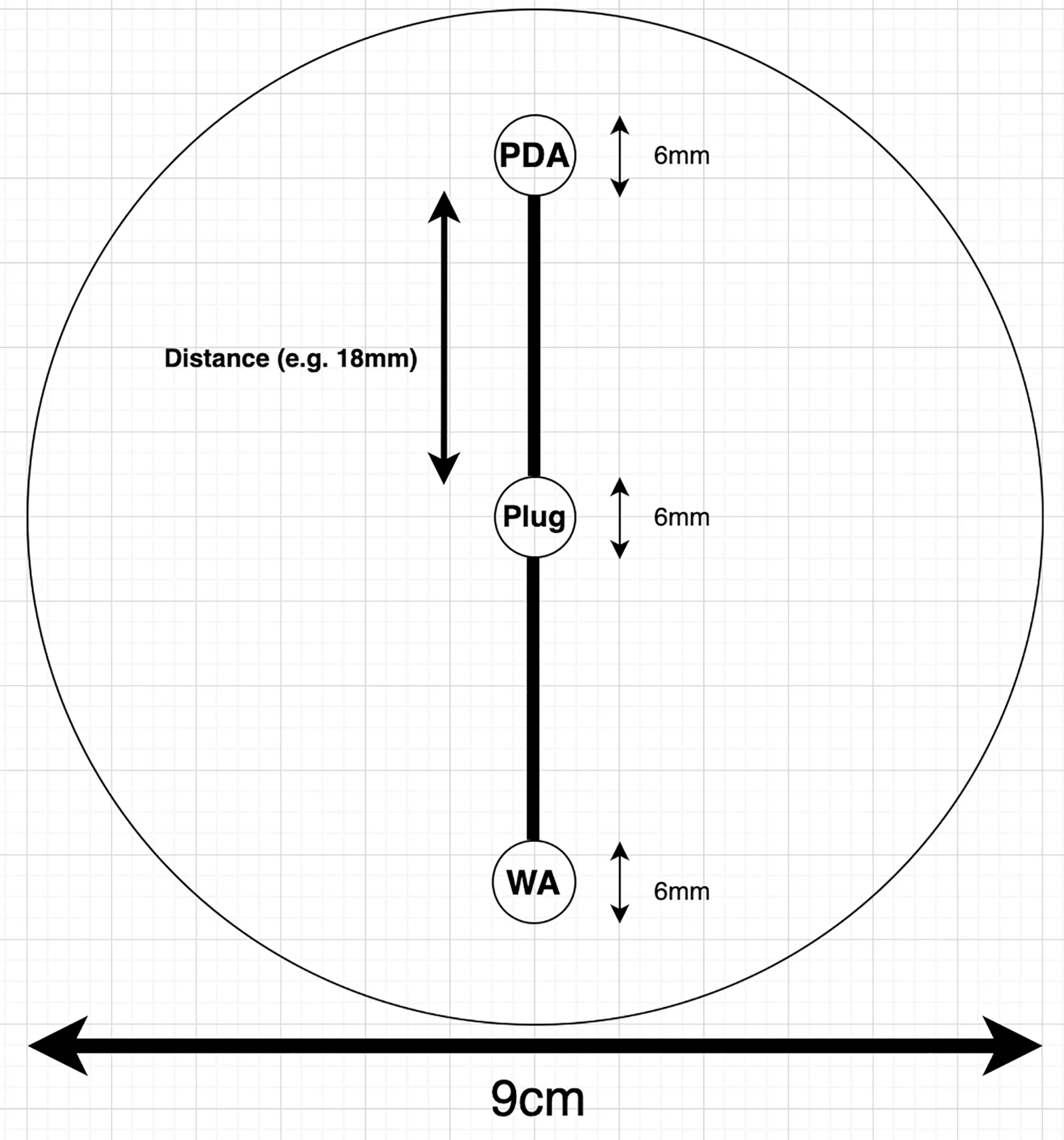
Placement of fungal plugs, WA, and PDA. The bidirectional setup was designed to avoid interference between treatments due to simple diffusion.

The initial experiments tested six fungal strains, and the core growth experiment focused on fungal strain B36DE6 (*Hypoxylon* sp., isolated from senescent tissue of *Sphagnum junghuhnianum*), as the other strains showed little or no preference for the food source. Full-length ITS sequences for B36DE6 is include in Data S1. A 6 mm fungal plug was extracted from a pre-cultured plate using a cork borer and transferred to solid growth medium. The solid medium consisted of 20 mL of sterilized medium containing 15 g/L agarose (Difco^™^ Agar; Becton, Dickinson and Company (BD), Franklin Lakes, NJ, USA), poured into 9 cm-diameter Petri dishes. Two types of food source plugs were prepared: Potato Dextrose Agar (PDA) plugs and Water Agar (WA) plugs. The WA plugs served as the control group. PDA plugs were prepared using a 1:50 dilution of Potato Dextrose Agar (Difco^™^ Potato Dextrose Broth; BD, Franklin Lakes, NJ, USA) according to standard protocol, containing 15 g/L agarose. Ten mL of PDA was poured into six cm-diameter Petri dishes, and 6 mm plugs were extracted and placed onto the solid medium as food sources. The inoculation plug was positioned at the center of each 9-cm Petri dish. Five experimental setups were established with varying distances (6, 12, 18, 24, and 30 mm) between the inoculation plug and food source plugs (PDA and WA plugs). Six replications were prepared for each distance treatment. The experimental setup is illustrated in Fig. 4.

A comprehensive grid search using the ABM was conducted to identify parameter combinations that matched the fungal growth pattern in this experiment. All key parameters, birth and death coefficients, repulsion intensity parameter (γ), attraction strength parameter (σ), were systematically searched within the model, and simulated results were compared with digital images captured throughout the experiment. The attracting strength parameter is used to determine the direction of growth of the model, instead of the actual migration on the solid agar plate. This enabled researchers to identify potential underlying growth and movement mechanisms, subsequently using these parameters to compare multiple microbes for potential applications.

The growth pattern was monitored and photographed at days 4, 7, 11, and 14 post-inoculation based on preliminary growth data. Digital images were analyzed using ImageJ software, where hyphal extension distances were measured along reference lines drawn from the center of each fungal plug to the corresponding food source plug. Measurements were performed in triplicate with the experimenter blinded to treatment conditions. Statistical analysis was conducted using two-way analysis of variance (ANOVA) to evaluate treatment effects across different distances and time points. In addition, Student’s t-tests with False Discovery Rate (FDR) correction were performed on a subset of results where fungal growth had not yet reached the food source.

### Implementation

The ABM developed in this study was implemented in the R programming environment (R Core Team, 2025). The package developed in this work is compatible with R version 4.0.0 or later. The S3 class system was implemented to define distinct agent classes, enabling polymorphic behavior and the incorporation of new agent types. The underlying simulation mechanism is thoroughly tested using the “testthat” package to ensure accuracy and reproducibility (Wickham, 2011). This package and all source code are publicly available https://github.com/ComputationalAgronomy/simulation_bacteria. The specific version used is available https://doi.org/10.5281/zenodo.17774654.

## Results

### Simulation validation analyses

**Figure 5:**
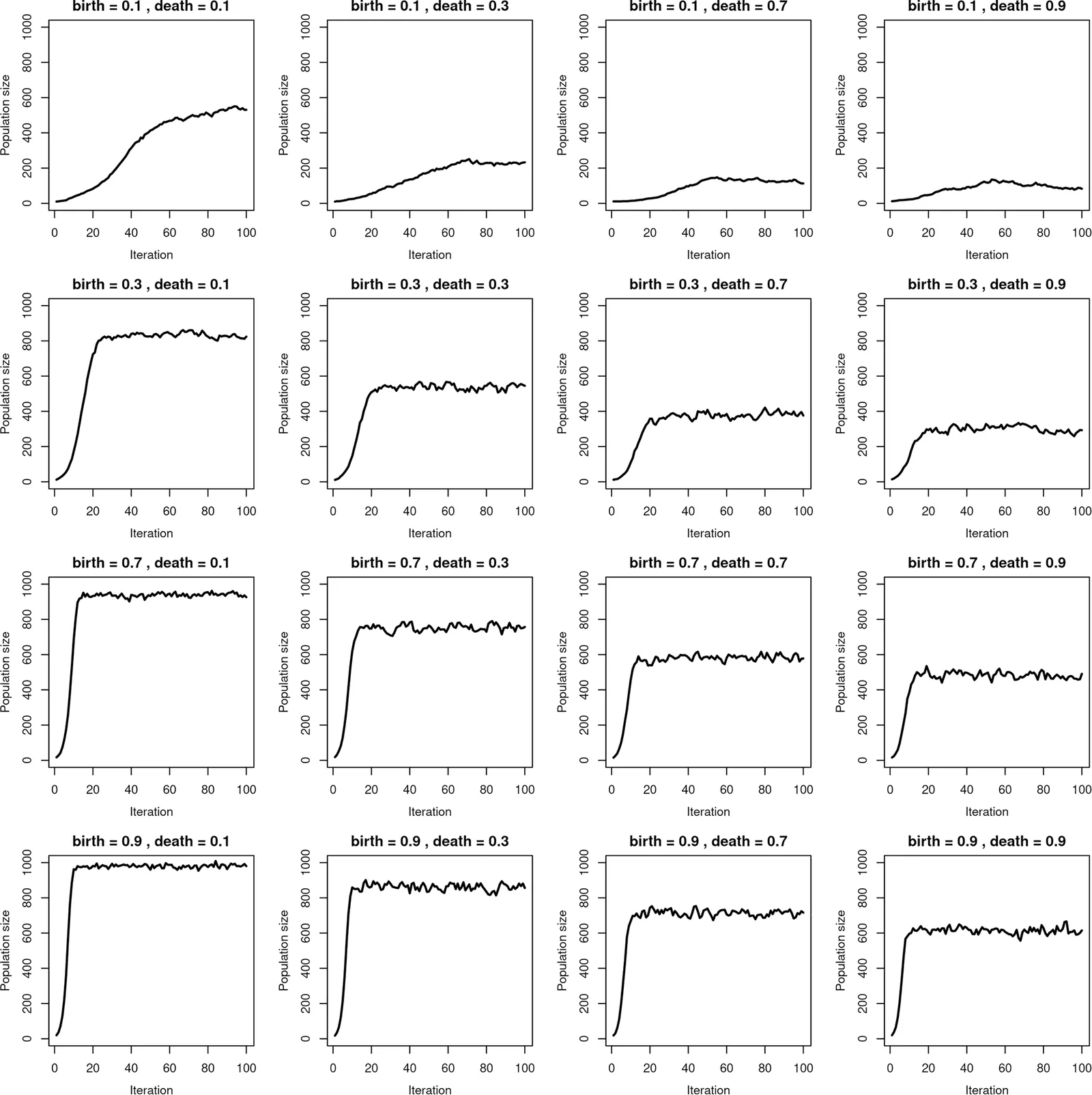
The effect of birth and death coefficients on the agent based model. Systematic grid search on the birth and death coefficients. The carrying capacity of 10,000 with an initial population of 10. A wider disparity between birth and death coefficients resulted in a larger population size.

A systematic grid search was conducted to validate the effect of birth and death coefficient combinations, and the results are illustrated in Fig. 5. The overall population dynamics reached equilibrium based on the balance between birth and death coefficients. The final population size oscillated around a threshold determined by the combination of these two coefficients. When the birth coefficient was high, the initial population growth phase was steep and reached the carrying capacity. On the other hand, when the death coefficient was high, the population declined towards extinction. In addition, the growth model with the birth coefficient only is tested and compared against the theoretical results from the differential equation. The results showed that the growth curve was consistent with expected values from the theoretical model (Fig. S1).

**Figure 6:**
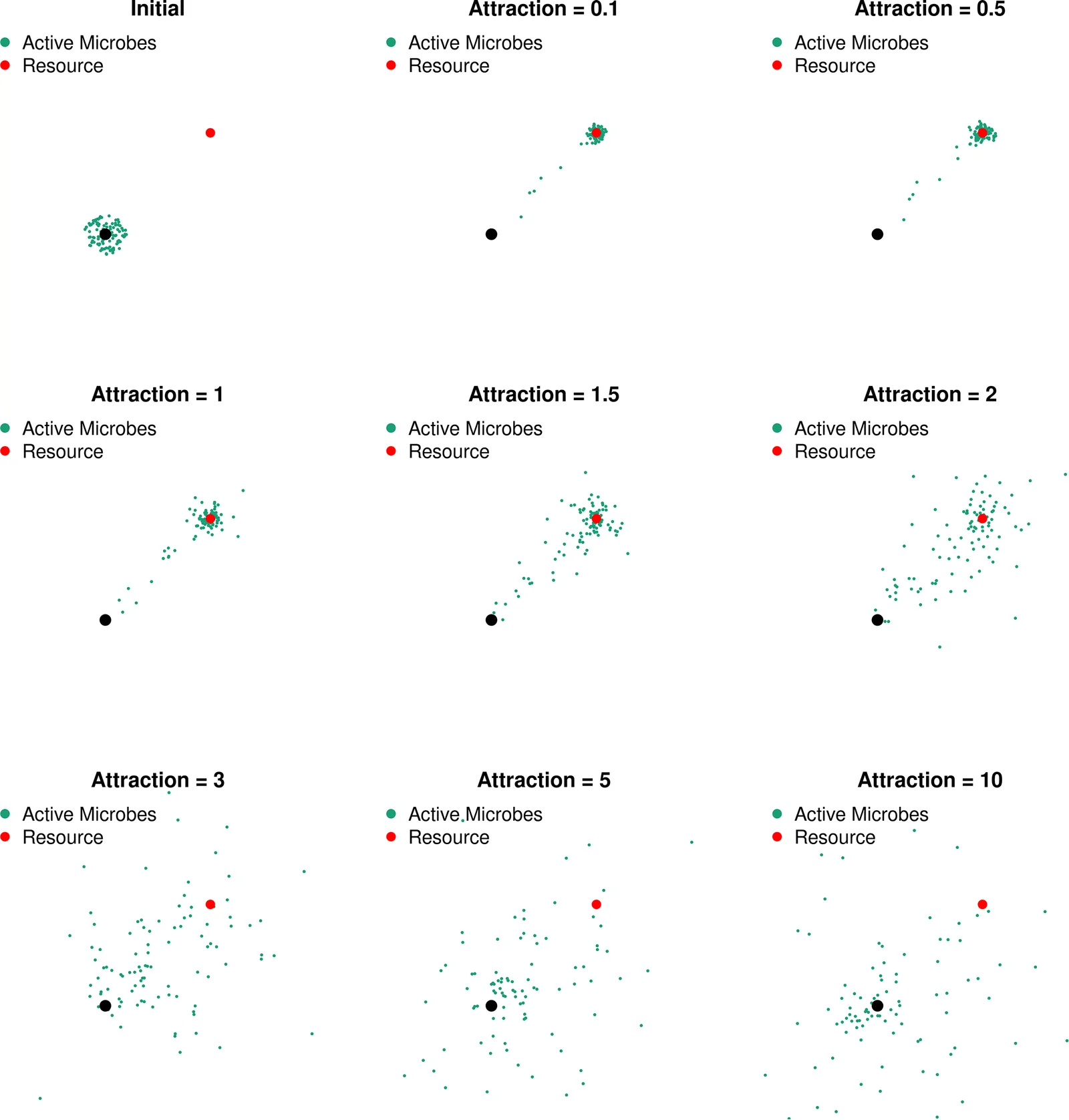
Simulation results for the attraction strength parameter (σ). The green dots represent the location of agents, the black dot represents the center of the initial location of all agents, and the red dot represents the resource. When the attraction strength parameter (σ) is large, agents appear to move randomly from the initial location. As the attraction strength decreased, agents moved and clustered around the resource.

The effect of the attraction strength parameter (σ) after 100 generations was illustrated in Fig. 6. The green dots represent the location of agents, the black dot represents the center of the initial location of all agents, and the red dot represents the resource. When the attraction strength parameter was large, the agents moved randomly and displayed no obvious patterns in their final location. As the parameter value decreases, agents show stronger attraction to the resource, and agents begin to aggregate around the location of the resource. When the parameter value drops to below 1, all agents form a tight cluster around the resource. The location of the agents can be tracked throughout generations, and individual agents’ movement speed determines the number of generations required for each agent to reach the final destination. To illustrate this process, 1,000 agents with attraction strength parameter σ = 2 were tracked for 100 generations. A snapshot was captured every 10 generations and presented in Fig. S2.

**Figure 7:**
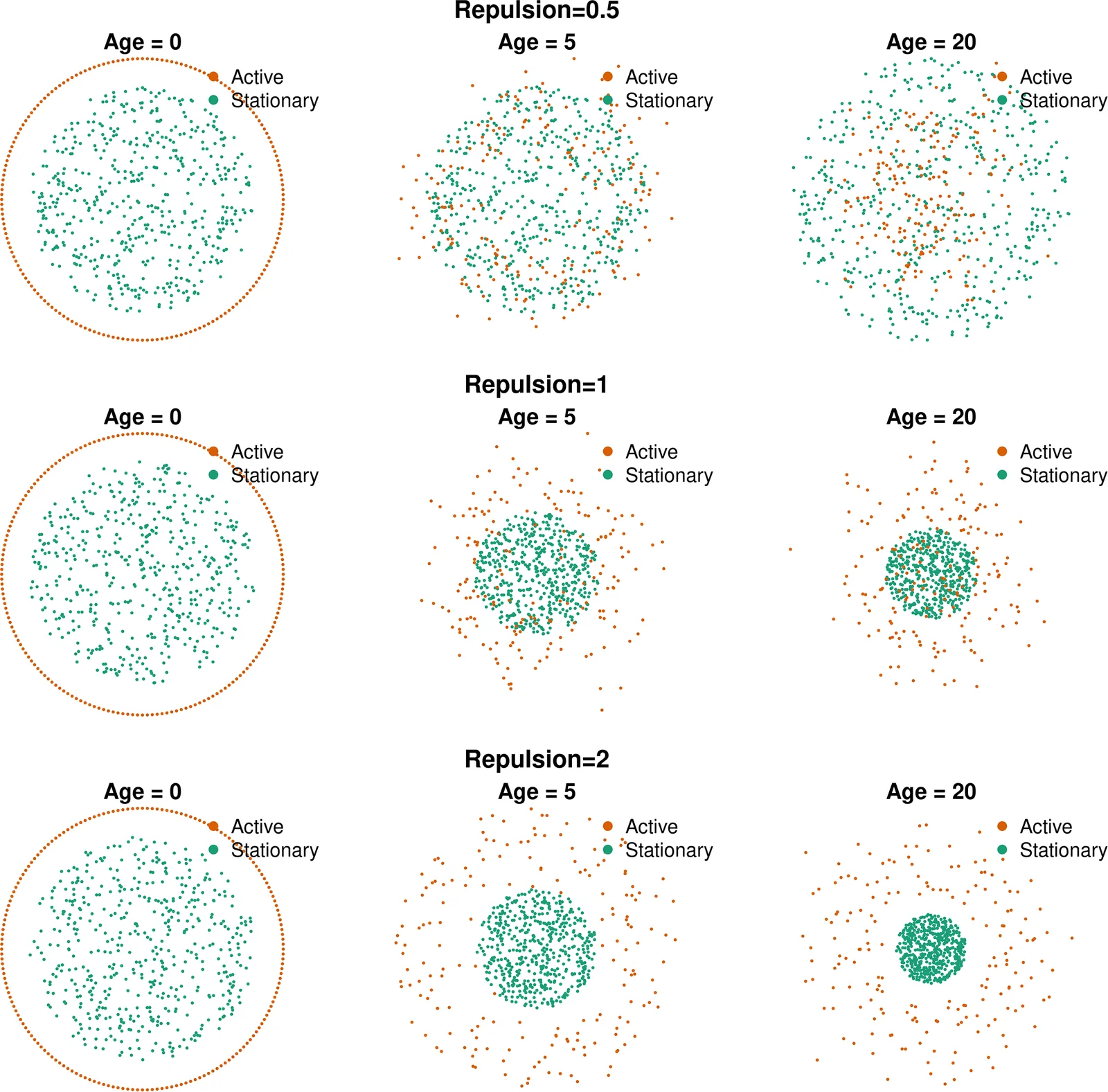
Simulation result for different repulsion intensity parameters (γ). When the repulsion intensity parameter (γ) is less than one (<1), it results in an attraction force, and the active agents (orange dots) move towards the center of the stationary agents (green dots). On the other hand, when γ is greater than one, the active agents move away from the stationary agent.

The effect of the repulsion intensity parameter (γ) is summarized in Fig. 7, where each row represents different parameter values, and different generations are displayed across columns. The active and mobile agents (blue dots) were positioned around the stationary agents (black dots) at the start of the simulation. As the simulation progressed, when γ was less than one, agents exhibited movement or growth toward other agents and clustered together with other background agents. When γ was greater than one, agents showed a repulsion effect from neighboring agents and attempted to move away from each other. When γ equaled one, there were no effects from the neighboring agents, and agents moved freely in all directions. The full simulation results are available in Fig. S3.

**Figure 8:**
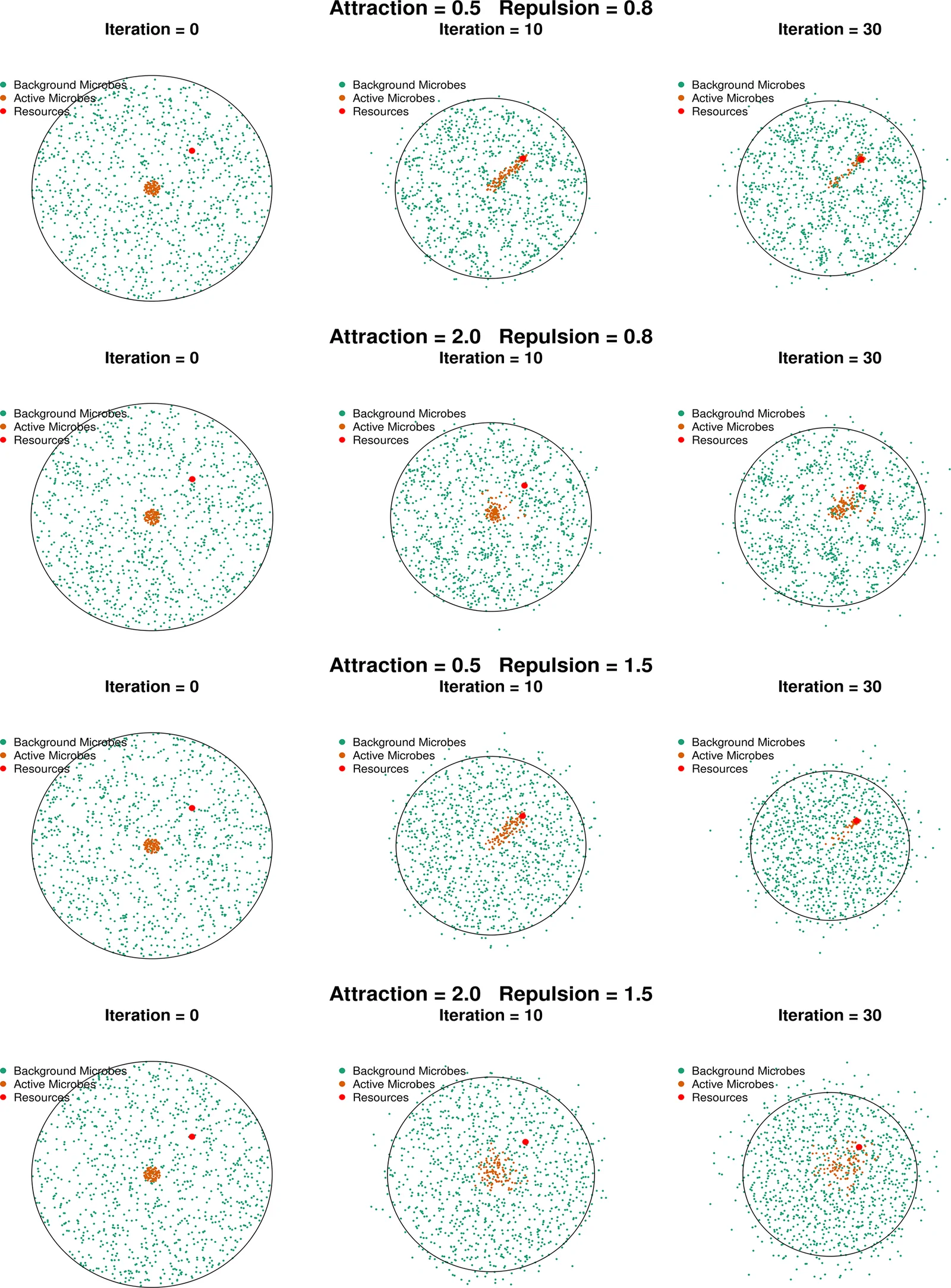
Combined simulation analyses for the bidirectional preference mechanism. The effects of the attraction strength parameter (σ) and repulsion intensity parameter (γ) on microbial behavior over time. Green dots represent background microbes with no resource preference, orange dots represent active microbes with resource preference, and the red dot represents the resource target. The black circle indicates the initial boundary of background microbes, which is not an actual physical boundary for the microbes. Snapshots are shown at multiple iterations to demonstrate that with the lower value of σ, active microbes cluster around the resource at a faster rate. The higher value of γ showed larger and loosely clustered areas due to the strong repulsion effect with the surrounding microbes.

The combined simulation validation analyses for the full model, which include both population dynamics and directional preference mechanisms, are summarized in Fig. 8. The green dots represent the background microbes with no preference for resources, and the initial boundary of the background microbes is represented by the black circle. The active microbes are represented by the orange dots, and they have a preference to move toward the resource, which is represented by the red dot. The full simulation results are available in Fig. S4.

The effect of multiple combinations of attraction strength parameter (σ) and repulsion intensity parameter (γ) is demonstrated in this analysis. Snapshots were captured for the initial population, and at iterations 0, 5, 10, 20, and 30. Results showed that the background microbes maintained a stable population and dispersed evenly in all directions for all scenarios. The effect of multiple combinations of attraction strength parameter (σ), and repulsion intensity parameter (γ) showed that when the value of σ for the active microbes (orange dots) was low, they actively moved toward the resource (red dot) and clustered around the resource within a few generations. When the attraction strength parameter was large, the resource attraction force was weaker, and it took more generations to cluster around the resource. The effect of the repulsion intensity parameter (γ) was difficult to observe. As the value of the parameter increased, the repulsion force from the background microbes slowed down the migration process of the active microbes and the active microbes formed a larger clustered area around the resource. To illustrate this subtle effect, additional simulations were conducted with some unrealistic and extreme values of the repulsion intensity parameter (γ), and results are plotted in Fig. S5. When the repulsion intensity parameter was at an extremely small value of 0.2, agents tended to cluster together. On the other hand, active agents (orange dots) formed a large cluster around the resource to avoid overcrowding with an extremely high parameter value of 10. The background microbes also dispersed further from the starting area as the black circle reduced to a smaller size on the plot to accommodate the position of these microbes.

### Biological validation analyses

Six fungal strains were cultured for 14 days, and the growth patterns were recorded on days 4, 7, 11, and 14. The growth distances were quantified from digital images using ImageJ software (Schneider, Rasband & Eliceiri, 2012). Nine strains exhibited symmetric growth, with comparable distances between the central plug and both the PDA plug and the water control plug. One strain (strain ID B36DE6, *Hypoxylon* sp.) demonstrated significant resource preference, and the growth rate toward the PDA plug is faster compared to the rate toward the water control. Some images were corrupted for setup with the distance 24 mm on Day 7, therefore, these data were removed from all analyses. Examples of the growth patterns for this strain at a 30 mm distance between the food source and inoculation plug across four sampling days are shown in Fig. S6. All raw images are available at https://doi.org/10.5281/zenodo.17595927.

**Figure 9:**
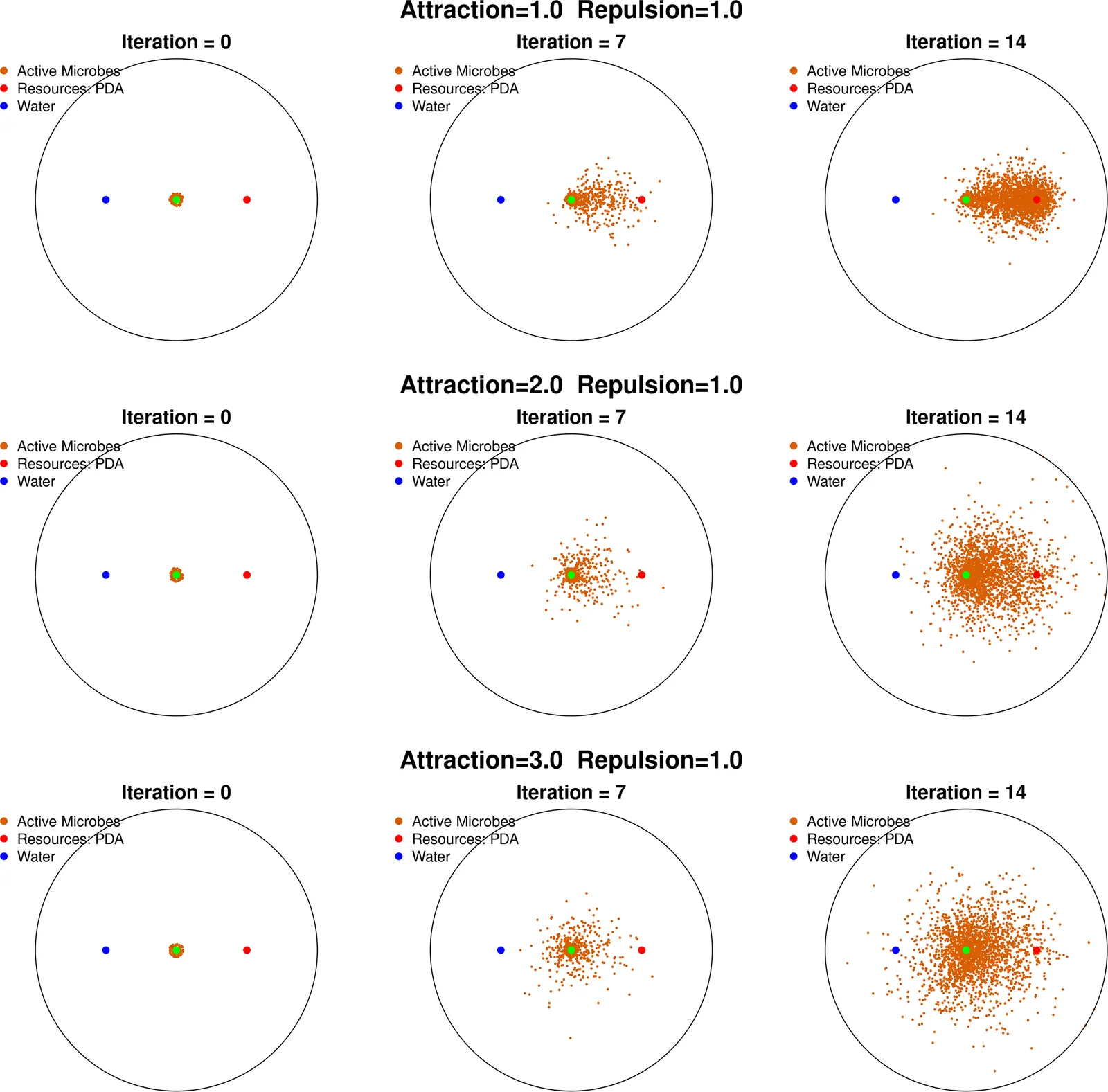
Simulated growth patterns for recovering the underlying parameter for the experimental fungal growth experiment. Simulation results show the growth pattern is comparable to the photos collected in the fungal growth experiment, when the attraction strength parameter σ is between 2.5 and 3.5. A low value of σ showed a very strong directional preference and skewed towards PDA, and a high value of σ is nearly indistinguishable between PDA and WA.

Simulation data are able to recreate a similar pattern based on the following parameter combination: Birth coefficient = 0.3, Death coefficient 0.1, repulsion intensity parameter γ = 1, and attraction strength parameter (σ) between 2 and 3. The simulated growth pattern was shown in Fig. 9, which demonstrated the model’s ability to capture directional microbial movement toward attractive resources, consistent with the objective of beneficial microbe navigation toward plant roots. The detection radius and angle were set to 1° and 45°, and these parameters have a limited effect on the overall growth pattern. Although the ABM does not perfectly reproduce the growth pattern, the model provides insight into the possible underlying strength of resource preferences. The attraction strength parameter (σ) is greater than 2, also suggesting that it has a relatively strong preference for the food source; hence, significant differences were observed between the PDA and WA plugs. Additional simulation result from the grid search are available in Fig. S7.

Two-way ANOVA was performed on each experimental setup and results showed significant differences in growth distance between PDA and water plugs for experimental setups with 6, 18, 24, and 30 mm distances. However, there were indications of interaction between treatment and day effects, as growth rate changed after reaching the food source plug. Individual t-tests revealed that there were no differences between treatments once they reached the food source plugs. ANOVA results are available in Table S1. Therefore, additional Student’s t-tests with FDR correction were performed on data collected prior to reaching the PDA or WA plugs (Table 1). Results showed significant growth difference between PDA and WA on six out of eight treatments, 18 mm Day 4 and Day 7, 24 mm Day 4, and 30 mm Day 7, 11, 14. This indicated a growth preference towards the PDA plugs over the WA plugs.

**Table 1:** Student’s t-test results comparing fungal growth distances between food source treatment (PDA *vs*. WA) prior to reaching food source plugs. *P*-values FDR-corrected for multiple comparisons.

	12 mm day 4	18 mm day 4	18 mm day 7	24 mm day 4	30 mm day 4	30 mm day 7	30 mm day 11	30 mm day 14
*P*-value (unadjusted)	0.0484	0.0121	<0.001	0.0062	0.176	<0.001	<0.001	<0.001
*P*-value (FDR)	0.0553	0.0162	<0.001	0.0099	0.176	<0.001	<0.001	<0.001

**Note:**

FDR, False Discovery Rate.

## Discussion

A generic ABM framework was developed and validated to simulate microbial behavior and spatial dynamics in soil systems, and focus on understanding how inoculated microbes navigate toward plant roots under agricultural scenarios. This model incorporated population dynamics and directional preference mechanisms of microbes, which were both essential mechanisms for microbial behavior. The simulation results demonstrated that the model was able to capture different aspects of microbial behavior and interactions with the surrounding environment.

The population dynamics simulations successfully reproduced the growth patterns and closely matched theoretical differential equation results. This validation demonstrated that the ABM framework adequately captured fundamental microbial growth mechanisms while providing greater flexibility than mathematical models. The ABM also allows dynamic parameter specification, and researchers to specify multiple coefficients at different stages of the simulation. For instance, the birth coefficient could be set to 0.2 at the first ten generations, and subsequently increase to 0.6 with the introduction of additional resources to the environment. The flexibility of this approach allows biologists to test and validate various scenarios without rewriting the differential equations for the population dynamics.

The directional preference mechanisms in this model are governed by two major factors: resource attraction and density-dependent interactions with surrounding microbes. This study aimed to determine how agricultural biologicals applied to soil migrate toward plant seeds or roots by modeling the attractiveness of plant exudates, chemotactic signals from plants, and the interactive effects between all microbes in the soil. The simulation results demonstrated that the attraction strength parameter (σ) effectively modulated microbial aggregation behavior around resource locations. This finding supports the idea that plants can attract microbes from a distance, and the extent of this attraction depends on the specific plant-derived substance (Schulz-Bohm et al., 2018). When σ < 1, agents formed tight clusters around resources, regardless of small variations in the value of the parameter, simulating the strong responses observed in rhizosphere colonization studies. This finding supports the hypothesis that plant root exudates can attract beneficial microbes from considerable distances, with attraction strength varying according to the different chemical compounds involved. The attraction strength was modeled using a normal distribution for movement probability, where the standard deviation σ modulated attraction strength. This approach provided a computationally efficient method for simulating gradient-following behavior without explicitly modeling chemical concentration fields, and maintaining biological realism while reducing computational complexity for large-scale population studies. The density factor captured repulsion effects that caused microbes to avoid overcrowded areas with potentially high resource competition and maintain spatial separation from competitors. The repulsion intensity parameter (γ) captured avoidance behaviors between microbes. Results showed that γ > 1 induced repulsion effects, causing microbes to maintain spatial distances from each other and avoid overcrowded areas. This was consistent with observations of bacterial biofilm dispersion (Rumbaugh & Sauer, 2020). Conversely, γ < 1 promoted aggregation behavior, while γ = 1 eliminated density effects entirely, allowing for systematic parameter isolation. The detection radius (ρ) and detection angle (α) parameters provided additional control over the spatial and directional preference. Our results suggested that small adjustments (*i.e*., ±10°) to the detection angle had minimal effects compared to the repulsion intensity parameter, indicating that the strength of density response may be more influential than the spatial precision of density detection in determining outcomes. However, these three factors interacted with each other, and the effect of each parameter could not be completely ignored. For example, when the detection angle was large but the detection radius was small, an agent could perceive the impact of density from a distance, rendering a larger value of γ less effective compared to scenarios with a smaller detection angle. The simulation results suggested that the model captured fundamental microbial behaviors rather than parameter-specific patterns. This provided a tool for general comparison between different potential microbial products.

The biological experiment demonstrates that microorganisms had a preference to grow toward the food source. Comparing the simulation results with real experiments, the model was able to identify a range of parameter combinations that could explain the overall growth patterns in the presence of attractive resources. Although most of the parameters in the model were relative values without absolute units, comparing these relative values can still provide insight into microbial behavior. For instance, the model estimated the parameters for a large number of microbes based on experimental data, and researchers could compare and rank these parameters to identify the most promising microbial candidates for the next phase of studies. Comparison between simulated results and experimental records is a challenging step. There are multiple factors that need to be considered, such as resolution of the image, matching dimensions between simulation and experiment. Unfortunately, there are no robust algorithms or reliable software to perform the comparison. The urgency of developing such algorithms will enhance the usability of the simulation results, and they can be more easily translated and compared to experimental images.

There are several existing models attempting to describe microbial movement processes. Some chemotaxis models have used the Keller-Segel model and differential equations to describe microbial movement and aggregation (Perthame, 2004; Romanczuk et al., 2008; Seyrich, Palugniok & Stark, 2019). Others use reaction-diffusion frameworks to characterize macroscopic behaviors such as spatial patterning and colony morphology (Mimura, Sakaguchi & Matsushita, 2000; Maslovskaya, Shuai & Kuttler, 2025; Bisi et al., 2026). These models provide detailed and precise mathematical descriptions of the underlying microbial movement processes. Nevertheless, they are often based on specific mathematical formulations that require detailed parameterization, including chemical gradients and other parameters that are challenging to measure in complex soil environments. Several existing ABM frameworks have demonstrated the power of individual-based approaches in capturing microbial community behaviors and interactions, with some focusing on intracellular processes, single-cell chemotaxis, and biofilm formation (Koshy-Chenthittayil et al., 2021; Nagarajan, Ni & Lu, 2022; Ni & Lu, 2022). The ABM presented here complements these models by providing a flexible framework specifically tailored to agricultural biological applications.

This model also provided a generic framework for expanding to include additional mechanisms of interest. There are other key mechanisms that could be incorporated with minimal or no modification to the framework. For example, the model could incorporate multiple resources of attraction for the microbes, or alter the attraction strength throughout the simulation. Another extension could incorporate multiple different types of interactions between inoculated microbes and surrounding background microbes (Macy & Willer, 2002; Wang et al., 2011). Additionally, integrating environmental factors such as temperature, pH, and moisture gradients into the model would enhance biological realism (Fravel, Olivain & Alabouvette, 2003; Aung, Jiang & He, 2018). Although these would make the model more realistic, in reality, it is also challenging or sometimes impractical to measure the moisture on a fine scale (*i.e*., every 5 cm) across the whole field. Therefore, these advanced mechanisms were left out of the current implementation, but represent important directions for future research.

The interactions between microorganisms in the soil were not limited to competing for space and resources. There were millions to billions of microorganisms in a gram of soil, and the complexity of all these interactions would be very challenging to model due to various underlying mechanisms, such as resource competition, chemical signaling, mutualistic partnerships, and predator-prey dynamics operating at different scales and timescales. This model simplified the three-dimensional complexity of soil environments down to a two-dimensional system, potentially making scenarios easier to understand. However, this oversimplified the spatial complexities of the soil systems, and did not take into account all possible interactions that could occur in real applications. Additionally, the assumption of fixed resource locations did not account for the dynamic nature of root exudate diffusion and temporal variation in chemical gradients. Despite these limitations, the model provides a useful platform for evaluating and comparing microbial candidates for agricultural biological applications.

## Conclusion

This study developed and validated an Agent-Based Model (ABM) to simulate microbial behavior in soil environments for agricultural biological applications. The model successfully addressed a knowledge gap in understanding how inoculated microbes navigate through complex soil environments and respond to plant derived chemical signals. The results of this study demonstrated several important key findings. The ABM successfully integrates a logistic based birth and death model for population dynamics with directional preference mechanisms, including resource attraction to plant exudates and density based repulsion from interactions with surrounding microorganisms. Furthermore, simulation results demonstrated that the ABM can reproduce theoretical population growth patterns and capture chemotactic responses. Key parameters, including birth and death coefficients, attraction strength parameter (σ), and repulsion intensity parameter (γ), effectively describe spatial patterns throughout the simulation. Laboratory validation experiments conducted on agar plates confirmed preferential growth toward nutrient sources, with observed growth patterns corresponding well to simulation results. Together, these findings establish the ABM as a reliable simulation tool for studying microbial behavior and plant-microbe interactions in agricultural systems. The current ABM framework provides a solid foundation for expanding simulation complexity, incorporating additional microbial behaviors and interactions with the environment. This provides a practical computational tool for researchers to bridge the gap between laboratory observations and underlying mechanisms. This enables researchers to target a range of different research questions and identify optimal strategies for developing effective agricultural biological products.

## Supplemental Information

10.7717/peerj.21641/supp-1 Supplemental Information 1 Validation of Simulated ABM growth curve compared against the theoretical growth model. The ABM simulated the growth curve 10 times under a range of birth coefficients and compared the results to the corresponding theoretical logistic growth curves. The growth curves from the ABM match closely to the theoretical curves, except when the birth coefficient is very low at 0.05. This is due to the stochastic fluctuations during the initial phase that cause the different growth trajectories later in the simulation.

10.7717/peerj.21641/supp-2 Supplemental Information 2 Snapshots of microbial agent movement across 100 generations. A population of 1,000 agents with attraction strength parameter σ = 2 was tracked over 100 generations, with snapshots captured every 10 generations to illustrate the progressive spatial dynamics. The green dots represent the location of agents, the black dot represents the center of the initial location of all agents, and the red dot represents the resource.

10.7717/peerj.21641/supp-3 Supplemental Information 3 Full simulation result for different repulsion intensity parameters (γ).

10.7717/peerj.21641/supp-4 Supplemental Information 4 Full simulation results for combining attraction strength parameter (σ) and repulsion intensity parameter (γ) for the bidirectional preference mechanism.

10.7717/peerj.21641/supp-5 Supplemental Information 5 Effect of extreme value of the repulsion intensity parameter (γ). Simulation results with extreme values of the repulsion intensity parameter to demonstrate the effect on the microbial dynamics. The green dots represent the background microbes with no preference for resources, the orange dots represent active microbes, the red dot indicates the resource, and the initial boundary of the background microbes is represented by the black circle. At the low level of repulsion (γ = 0.2), microbes cluster tightly together. At the high level of repulsion (γ = 10), the active microbes (orange) form a large scattered cluster around the resource, and the background microbes (green) spread out further from their initial location.

10.7717/peerj.21641/supp-6 Supplemental Information 6 Growth pattern of strain B36DE6 (days 4, 7, 11, 14). Setup: 30 mm between central plug and food sources (PDA left, water right).

10.7717/peerj.21641/supp-7 Supplemental Information 7 Full simulation results for growth patterns for recovering the underlying parameter for the experimental fungal growth experiment. Simulation results show the growth pattern is comparable to the photos collected in the fungal growth experiment, when the attraction strength parameter σ is between 2 and 3. A low value of σ showed a very strong directional preference and skewed towards PDA, and a high value of σ is nearly indistinguishable between PDA and WA.

10.7717/peerj.21641/supp-8 Supplemental Information 8 Two-way ANOVA summary for treatment (PDA *vs*. water agar), day, and interaction effects on growth at 6, 12, 18, 24, and 30 mm from the initial inoculation plug.

10.7717/peerj.21641/supp-9 Supplemental Information 9 Full-length ITS sequences for B36DE6 (Hypoxylon sp., isolated from senescent tissue of *Sphagnum junghuhnianum*).

## Additional Information and Declarations

### DNA Deposition

The following information was supplied regarding the deposition of DNA sequences:

The sequence data are available at NCBI under accession number PZ725222.

### Data Availability

The following information was supplied regarding data availability:

The code is available at GitHub and Zenodo:

- https://github.com/ComputationalAgronomy/simulation_bacteria

- Yuan, S., & Steven Wu. (2025). ComputationalAgronomy/simulation_bacteria: manuscript (manuscript). Zenodo. https://doi.org/10.5281/zenodo.17774654

The data is available at Zenodo: Wu, S. H. (2025). Raw Data for the agent-modes model validation. Zenodo. https://doi.org/10.5281/zenodo.17595927

The sequence data is available in the Supplemental Files.

## References

[ref-1] Abu-Ashour J, Joy DM, Lee H, Whiteley HR, Zelin S (1994). Transport of microorganisms through soil. Water, Air, and Soil Pollution.

[ref-2] Ahmad A, Javad S, Iqbal S, Shahid T, Naz S, Shah AA, Shaffique S, Gatasheh MK (2024). Efficacy of soil drench and foliar application of iron nanoparticles on the growth and physiology of *Solanum lycopersicum* L. exposed to cadmium stress. Scientific Reports.

[ref-3] An L (2012). Modeling human decisions in coupled human and natural systems: review of agent-based models. Ecological Modelling.

[ref-4] Araújo RG, Chavez-Santoscoy RA, Parra-Saldívar R, Melchor-Martínez EM, Iqbal HMN (2023). Agro-food systems and environment: sustaining the unsustainable. Current Opinion in Environmental Science & Health.

[ref-5] Aung K, Jiang Y, He SY (2018). The role of water in plant–microbe interactions. The Plant Journal.

[ref-6] Bamdad H, Papari S, Lazarovits G, Berruti F (2022). Soil amendments for sustainable agriculture: microbial organic fertilizers. Soil Use and Management.

[ref-7] Bente K, Mohammadinejad S, Charsooghi MA, Bachmann F, Codutti A, Lefèvre CT, Klumpp S, Faivre D (2020). High-speed motility originates from cooperatively pushing and pulling flagella bundles in bilophotrichous bacteria. eLife.

[ref-8] Bisi M, Cusseddu D, Soares AJ, Travaglini R (2026). Reaction–diffusion systems from kinetic models for bacterial communities on a leaf surface. Journal of Nonlinear Science.

[ref-9] Bonabeau E (2002). Agent-based modeling: methods and techniques for simulating human systems. Proceedings of the National Academy of Sciences of the United States of America.

[ref-10] Buchanan RL, Whiting RC, Damert WC (1997). When is simple good enough: a comparison of the Gompertz, Baranyi, and three-phase linear models for fitting bacterial growth curves. Food Microbiology.

[ref-11] Canarini A, Kaiser C, Merchant A, Richter A, Wanek W (2019). Root exudation of primary metabolites: mechanisms and their roles in plant responses to environmental stimuli. Frontiers in Plant Science.

[ref-12] Chaudhary P, Singh S, Chaudhary A, Sharma A, Kumar G (2022). Overview of biofertilizers in crop production and stress management for sustainable agriculture. Frontiers in Plant Science.

[ref-13] Cuevas E (2020). An agent-based model to evaluate the COVID-19 transmission risks in facilities. Computers in Biology and Medicine.

[ref-14] Du H, Xu W, Zhang Z, Han X (2021). Bacterial behavior in confined spaces. Frontiers in Cell and Developmental Biology.

[ref-15] Elnahal ASM, El-Saadony MT, Saad AM, Desoky E-SM, El-Tahan AM, Rady MM, AbuQamar SF, El-Tarabily KA (2022). The use of microbial inoculants for biological control, plant growth promotion, and sustainable agriculture: a review. European Journal of Plant Pathology.

[ref-16] Fravel D, Olivain C, Alabouvette C (2003). *Fusarium oxysporum* and its biocontrol. New Phytologist.

[ref-17] Hibbing ME, Fuqua C, Parsek MR, Peterson SB (2010). Bacterial competition: surviving and thriving in the microbial jungle. Nature Reviews Microbiology.

[ref-18] Kerr CC, Stuart RM, Mistry D, Abeysuriya RG, Rosenfeld K, Hart GR, Núñez RC, Cohen JA, Selvaraj P, Hagedorn B, George L, Jastrzębski M, Izzo AS, Fowler G, Palmer A, Delport D, Scott N, Kelly SL, Bennette CS, Wagner BG, Chang ST, Oron AP, Wenger EA, Panovska-Griffiths J, Famulare M, Klein DJ (2021). Covasim: an agent-based model of COVID-19 dynamics and interventions. PLOS Computational Biology.

[ref-19] Koshy-Chenthittayil S, Archambault L, Senthilkumar D, Laubenbacher R, Mendes P, Dongari-Bagtzoglou A (2021). Agent based models of polymicrobial biofilms and the microbiome—a review. Microorganisms.

[ref-20] König S, Vogel H-J, Harms H, Worrich A (2020). Physical, chemical and biological effects on soil bacterial dynamics in microscale models. Frontiers in Ecology and Evolution.

[ref-21] Lobry JR, Flandrois JP, Carret G, Pave A (1992). Monod’s bacterial growth model revisited. Bulletin of Mathematical Biology.

[ref-22] Macy MW, Willer R (2002). From factors to actors: computational sociology and agent-based modeling. Annual Review of Sociology.

[ref-23] Mahanty T, Bhattacharjee S, Goswami M, Bhattacharyya P, Das B, Ghosh A, Tribedi P (2017). Biofertilizers: a potential approach for sustainable agriculture development. Environmental Science and Pollution Research.

[ref-24] Maslovskaya A, Shuai Y, Kuttler C (2025). The Allen–Cahn-based approach to cross-scale modeling bacterial growth controlled by quorum sensing. Mathematics.

[ref-25] Mimura M, Sakaguchi H, Matsushita M (2000). Reaction–diffusion modelling of bacterial colony patterns. Physica A: Statistical Mechanics and its Applications.

[ref-26] Mishra DP, Dash DD (2014). Rejuvenation of biofertiliser for sustainable agriculture economic development (SAED). Consilience.

[ref-27] Mitchell DA, von Meien OF, Krieger N, Dalsenter FDH (2004). A review of recent developments in modeling of microbial growth kinetics and intraparticle phenomena in solid-state fermentation. Biochemical Engineering Journal.

[ref-28] Nagarajan K, Ni C, Lu T (2022). Agent-based modeling of microbial communities. ACS Synthetic Biology.

[ref-29] Ni C, Lu T (2022). Individual-based modeling of spatial dynamics of chemotactic microbial populations. ACS Synthetic Biology.

[ref-30] Perthame B (2004). PDE models for chemotactic movements: parabolic, hyperbolic and kinetic. Applications of Mathematics.

[ref-31] R Core Team (2025). R: a language and environment for statistical computing.

[ref-32] Rajput RS, Ram RM, Vaishnav A, Singh HB, Satyanarayana T, Das SK, Johri BN (2019). Microbe-based novel biostimulants for sustainable crop production. Microbial Diversity in Ecosystem Sustainability and Biotechnological Applications: Soil & Agroecosystems.

[ref-33] Rehman A, Farooq M, Lee D-J, Siddique KHM (2022). Sustainable agricultural practices for food security and ecosystem services. Environmental Science and Pollution Research.

[ref-34] Romanczuk P, Erdmann U, Engel H, Schimansky-Geier L (2008). Beyond the Keller-Segel model. The European Physical Journal Special Topics.

[ref-35] Rumbaugh KP, Sauer K (2020). Biofilm dispersion. Nature Reviews Microbiology.

[ref-36] Santos F, Melkani S, Oliveira-Paiva C, Bini D, Pavuluri K, Gatiboni L, Mahmud A, Torres M, McLamore E, Bhadha JH (2024). Biofertilizer use in the United States: definition, regulation, and prospects. Applied Microbiology and Biotechnology.

[ref-37] Schneider CA, Rasband WS, Eliceiri KW (2012). NIH image to ImageJ: 25 years of image analysis. Nature Methods.

[ref-38] Schulz-Bohm K, Gerards S, Hundscheid M, Melenhorst J, de Boer W, Garbeva P (2018). Calling from distance: attraction of soil bacteria by plant root volatiles. The ISME Journal.

[ref-39] Seyrich M, Palugniok A, Stark H (2019). Traveling concentration pulses of bacteria in a generalized Keller–Segel model. New Journal of Physics.

[ref-40] Shahwar D, Mushtaq Z, Mushtaq H, Alqarawi AA, Park Y, Alshahrani TS, Faizan S (2023). Role of microbial inoculants as bio fertilizers for improving crop productivity: a review. Heliyon.

[ref-41] Sharma N (2014). Biologicals. Biological Controls for Preventing Food Deterioration.

[ref-42] Song H-S, Cannon WR, Beliaev AS, Konopka A (2014). Mathematical modeling of microbial community dynamics: a methodological review. Processes.

[ref-43] Torsvik V, Øvreås L (2002). Microbial diversity and function in soil: from genes to ecosystems. Current Opinion in Microbiology.

[ref-44] Tsoularis A, Wallace J (2002). Analysis of logistic growth models. Mathematical Biosciences.

[ref-45] Wang X, Pan Q, Chen F, Yan X, Liao H (2011). Effects of co-inoculation with arbuscular mycorrhizal fungi and rhizobia on soybean growth as related to root architecture and availability of N and P. Mycorrhiza.

[ref-46] Wickham H (2011). testthat: get started with testing. The R Journal.

[ref-47] Will M, Groeneveld J, Frank K, Muller B (2020). Combining social network analysis and agent-based modelling to explore dynamics of human interaction: a review. Socio-Environmental Systems Modelling.

[ref-48] Yang L, Qian X, Zhao Z, Wang Y, Ding G, Xing X (2024). Mechanisms of rhizosphere plant-microbe interactions: molecular insights into microbial colonization. Frontiers in Plant Science.

[ref-49] Zhu Y-G, Gillings M, Simonet P, Stekel D, Banwart S, Penuelas J (2017). Microbial mass movements. Science.

[ref-50] Zwietering MH, Jongenburger I, Rombouts FM, van ’t Riet K (1990). Modeling of the bacterial growth curve. Applied and Environmental Microbiology.

