# Supplementary material for: Agent-based model for simulating microbial movement with a preferred target for agricultural biologicals
Source: PeerJ. 2026 Sep 8;14:e21641. doi: 10.7717/peerj.21641 (PMC13564397; doi:10.7717/peerj.21641)
Supplement: Supplementary file 1 [file peerj-14-21641-s008.docx]

Supplementary Table S1: Two-way ANOVA summary for treatment (PDA vs. water agar), day, and interaction effects on growth at 6, 12, 18, 24, and 30 mm from the initial inoculation plug.

| Food Source distance (mm) | Source | Df | Sum Sq | Mean Sq | F value | Pr(>F) |
| --- | --- | --- | --- | --- | --- | --- |
| 6 | Trt | 1 | 4.5326 | 4.5326 | 9.1197 | 0.00439 |
| 6 | Day | 3 | 1.2734 | 0.4245 | 0.8541 | 0.47276 |
| 6 | Trt:Day | 3 | 0.9598 | 0.3199 | 0.6437 | 0.59147 |
| 6 | Residuals | 40 | 19.8802 | 0.497 |  |  |
|  |  |  |  |  |  |  |
| 12 | Trt | 1 | 1.997 | 1.997 | 2.2868 | 0.13834 |
| 12 | Day | 3 | 252.096 | 84.032 | 96.24 | < 2e-16 |
| 12 | Trt:Day | 3 | 6.441 | 2.147 | 2.4589 | 0.07675 |
| 12 | Residuals | 40 | 34.926 | 0.873 |  |  |
|  |  |  |  |  |  |  |
| 18 | Trt | 1 | 18.13 | 18.13 | 4.2624 | 0.04549 |
| 18 | Day | 3 | 1241.54 | 413.85 | 97.2954 | < 2e-16 |
| 18 | Trt:Day | 3 | 29.8 | 9.93 | 2.3354 | 0.0883 |
| 18 | Residuals | 40 | 170.14 | 4.25 |  |  |
|  |  |  |  |  |  |  |
| 24 | Trt | 1 | 42.68 | 42.68 | 21.322 | 6.84E-05 |
| 24 | Day | 2 | 2781.45 | 1390.73 | 694.713 | < 2.2e-16 |
| 24 | Trt:Day | 2 | 12.82 | 6.41 | 3.201 | 0.05495 |
| 24 | Residuals | 30 | 60.06 | 2 |  |  |
|  |  |  |  |  |  |  |
| 30 | Trt | 1 | 123.7 | 123.71 | 132.242 | 2.96E-14 |
| 30 | Day | 3 | 3501.5 | 1167.15 | 1247.611 | < 2.2e-16 |
| 30 | Trt:Day | 3 | 40.8 | 13.6 | 14.542 | 1.49E-06 |
| 30 | Residuals | 40 | 37.4 | 0.94 |  |  |
